# Hospital Saturday, the Truck Act and Workmen's Contributions

**Published:** 1914-07-04

**Authors:** 


### The Hospital

The Workers' Newspaper of Administrative Medicine and Institutional Life, Administration, National Insurance and Health.

No. 1463, Vol. LVI. SATURDAY, JULY 4, 1914. PRICE ONE PENNY.

### HOSPITAL SATURDAY, THE TRUCK ACTS AND WORKMEN'S CONTRIBUTIONS.

The June issue of *The Hospital Saturday Fund Journal* calls attention to the fact that there are people who would contribute to the Fund but do not for reasons beyond their control. The Journal properly claims for every one the right to give voluntarily to this Fund who may so desire. The difficulty of non-givers consists in the absence of an opportunity to hand in their gifts at the pay-table where they are employed. They object to a system which prevails in some shops of handing portions of such moneys to funds or institutions other than the Hospital Saturday Fund, which latter entitles them to benefits. These benefits are available to every member of the industrial classes who may contribute to the Fund, and their extent may be gathered from a statement which appears in another column.

We publish this week an exhaustive and very interesting paper by Mr. William Straker, corresponding secretary Northumberland Miners' Association, on a proposed amendment of the Truck Acts, which he properly holds may seriously affect the voluntary hospitals. These Acts do not apparently interest materially the voluntary hospitals in the Midlands and South of England, but in the North of England and in Scotland, should the Government not accept the warning given by Mr. Straker, it is held that several of the more important voluntary hospitals may be so crippled, financially, as to render it impossible for the managers to continue their present useful work with adequacy and efficiency, if at all. That this is the fact will become apparent from the figures of the volume of contributions by the workmen as we give them in this article. Mr. Straker boldly contends that if such contributions are "practically compulsory . . . it is the greatest compliment to the unseen influence of human goodness. If right were not to continue stronger than wrong, if goodness were to cease to be more powerful than evil, wrong and evil would triumph, and all humanitarian institutions, such as hospitals for the sick . . . . would perish. . . . I am absolutely convinced that there are a number of people who must be compelled to do their duty and contribute to a common fund for a common purpose for their own good and the good of society, otherwise civilised society could not exist."

Speaking of the subscriptions of working men to the Royal Victoria Infirmary, Newcastle-on-Tyne, in 1913, which amounted to over £20,000, Mr. Straker declares "It would be impossible to get at the most more than 50 per cent. of this by any other means than by deduction from wages at the pay office." This is proved, for when the Coal Mines Act 1911, which gave the right to the miners to receive weekly instead of fortnightly payments, was adopted, a few colliery owners refused to make deductions for the infirmary on the ground of the extra office work it entailed. The miners tried to induce the managers to make the deductions without success, and in consequence contributions from the workshops affected were reduced to the extent of 75 per cent. Mr. Straker maintained this result is not because workmen do not want to contribute, but because of the lack of necessary organisation for the success of any business. It cannot fail to be of importance and interest to hospital managers and the public generally throughout the country to have the striking evidence we have just given that in the great industrial centres, and also in London itself, workmen's contributions must be organised on business lines or they cannot succeed. The testimony of Mr. Straker strikingly confirms the justice of the contentions of the London Hospital Saturday Fund managers.

In this connection it may be well, as showing the actual relation which workpeople's contributions bear to voluntary hospital revenue, to set forth the extent to which the artisan classes contributed to 165 of the principal voluntary hospitals in the United Kingdom in 1912, the latest year for which complete figures are available. The workpeople contribute very little to voluntary hospitals in Ireland, such gifts being confined, so far as our information goes, mainly to Belfast. The Dublin hospitals, for instance, appear to have received nothing from this source, whilst workpeople's contributions in the rest of Ireland, excluding Belfast, amounted to £22, contributed to St. John's Hospital, Limerick. In Belfast workpeople's contributions were £4,957, in addition to a sum of £1,033 raised by the Hospital Saturday Fund, making a total of £5,990 from artisans. What have the workpeople of the rest of Ireland to urge in defence of their abstention from gifts to hospitals? In relation to the remaining portions of the United Kingdom it is interesting to note a few facts. First of all, the contributions from workpeople in London are derived mainly from the Hospital Saturday Fund. In Scotland Hospital Saturday is practically unknown; the workpeople's contributions are organised by several of the greater hospitals, to whose funds the artisans contribute directly. In the provinces upwards of five-fourteenths of the workpeople's contributions were derived from the Hospital Saturday Funds and upwards of nine-fourteenths directly from the workshops. We find that in 1912 the London Hospital Saturday Fund gave £17,684 to sixty-five London hospitals, whilst the workpeople's contributions were only £2,473. In the provinces the workpeople contributed directly to the voluntary hospitals £93,505, and the Hospital Saturday Fund £51,958. In Scotland the workpeople gave directly to the hospitals £34,764. We hope it may prove instructive and interesting to hospital managers throughout the country to have these facts in detail. The total sum raised in the United Kingdom from the sources in question in 1912 amounted to £206,521. Each separate organisation is constructed to some extent at any rate to meet local circumstances. Success or failure is largely governed by the ability and zeal which characterise the management of these funds and their direction each year.

